# Recruitment of Healthcare Staff to Social Network Studies: A Case Study Exploring Experiences, Challenges, and Considerations

**DOI:** 10.3390/ijerph15122778

**Published:** 2018-12-07

**Authors:** Aoife De Brún, Eilish McAuliffe

**Affiliations:** School of Nursing, Midwifery and Health Systems, University College Dublin, Dublin 4, Ireland; eilish.mcauliffe@ucd.ie

**Keywords:** social network analysis, recruitment, sampling, health systems research

## Abstract

Social network analysis (SNA) is a term that describes a set of methodologies to understand and depict social relations or ties. SNA is different from other research methods in several ways that have important ethical implications, as well as specific considerations for study design. Recruitment of participants and attrition during the study, where there are several data collection time points, pose significant challenges. Furthermore, there are implications of non-participation in studies, whereby gaps in network maps may result in an inaccurate representation of how a network is working and this, in turn, means the results may be of lesser value in terms of informing policy and practice. Given the widely noted challenge of recruiting healthcare staff to research, this paper adopts a case study approach to discussing considerations for researchers, as well as offering recommendations and insights from our own research and from the published literature about how to tackle these issues. This paper examines data sourcing, decision-making about defining the network for data collection, and ethical considerations and their implications for the recruitment of healthcare staff to social network studies. We use a case study example exploring leadership in a hospital group network to illustrate techniques and challenges in the recruitment of healthcare staff.

## 1. Introduction

Social network analysis (SNA) is a term that describes a set of methodologies to understand and depict social relations or ties [1]. Through posing specific questions to a group of respondents, the method enables researchers to make the relational patterns between individuals, events or organizations explicit. Typically, these relations are visually mapped and understood using social network metrics to examine the nature and structure of the network [2]. SNA has been employed in many domains, including health and health services research [3]. However, SNA is different from other research methods in several ways that have important ethical implications, as well as specific considerations for study design and dissemination of results. Recruitment of participants and attrition during the study, where there are several data collection timepoints pose significant challenges. The potential impact of these challenges on the trustworthiness of results and conclusions when data sets are incomplete is a key consideration for researchers employing SNA methods. Given the widely noted challenge of recruiting healthcare staff to research [4,5], this paper discusses considerations for researchers, as well as offering recommendations and insights from our own research and from the published literature about how to tackle these issues. This paper examines data sourcing, decision-making about defining the network for data collection, and ethical considerations and their implications for the recruitment of healthcare staff to social network studies.

The choice of data sources can have implications for completeness of data and for recruitment of participants. The decision regarding the most appropriate source of data in network research will depend on the research question of interest, and on the researcher’s access to, and potentially the ability to recruit, participants. Network data may be collected as primary data, i.e., collected by the researcher for the purpose of addressing specific questions, or as secondary data, where existing records or databases are re-examined to address questions of interest. The latter is often simpler and quicker to collect but imposes considerable constraints on the types of relations and network questions that may be studied. While paper-based archival data has traditionally been used as a secondary data source in SNA, with the increasing availability of big data sets (such as data collated via social media sites), there have been several examples of these new sources as creating opportunities for the study of social networks [6]. Clearly, the selection of the data source will have implications for whether participant recruitment is required, but there may be a trade-off in terms of potentially sacrificing data relevance and limiting options for analysis against a quicker and more efficient means of gathering data. For example, if patient records were used as a secondary data source, it may be possible to explore which healthcare team members most frequently worked with each other in direct patient care. However, without input from participants, one would not be able to address more specific questions concerning informal communication or advice-seeking outside of documented evidence of collaboration for patient care. Primary data collection from the network members of interest has the advantage of allowing for a more in-depth understanding of what may be influencing the ties or relations between members, identifying the factors influencing or dictating those close formal working relationships. Thus, the research question and data source will determine whether participant recruitment is required. 

The data source selected will also have implications for other decisions that will inform recruitment strategies. One major consideration is how to define and place boundaries on a network of interest. This is particularly challenging in healthcare settings where there are rarely tightly defined groups that are relatively isolated from external influence. Indeed, in healthcare as in most complex, open systems, teams operate within multi-team systems, which may all operate with, at least, some degree of interdependence in working towards to superordinate goal of the organization [7]. Furthermore, healthcare teams often have fuzzy boundaries whereby individuals are constantly rotating through teams during training and boundaries are highly permeable so that additional expertise may be introduced to the team on an ad-hoc basis when required [8]. Borgatti et al. [9] advise that if a research question does not allow you to restrict the network, a personal-network, or ‘ego-centric’ research design may be adopted. Decisions will still need to be made regarding who your respondents will be, but a random sample could be drawn from the population of interest. In contrast, a whole network study design attempts to include all individuals within a well-defined network boundary, for instance, all staff members working in an emergency department [10]. Failure to include all individuals or organizations in the network or the inclusion of non-members may constitute a threat to the validity of a study. However, choosing an artificial boundary is not necessarily a threat to the validity of the research design but may be appropriate in the specific context of a given study [9]. 

Once decisions regarding data sources to address the research question(s) and network boundaries have been considered, the next step involves participant recruitment using appropriate methods. Recruitment and sampling strategies will depend on whether an ego-centric or whole-network approach has been adopted. In a whole-network approach, all relevant members of a group or team are invited to take part in the study. This is known as a census approach. Alternatively, convenience sampling approaches, such as ego-centric or respondent-driven sampling methods may be used to collect data. Snowball sampling is a common approach adopted, where the researchers start with some identified participants (i.e., initial ‘seeds’) and further network members of interest are revealed through the responses of those initial participants who provide names and contact details of others [11]. These identified persons or organizations are then invited to participate in the study. However, this can be challenging when research may be sensitive in nature. Targeted location sampling is another technique which often includes initial mapping of the population with interviews with individuals at relevant sites [11]. 

The nature of the research topic, the type of questions posed, how the data will be used and disseminated will have implications for successful participant recruitment. For instance, in healthcare teams where questions are relevant to work, some participants may feel vulnerable in their role or exposed to criticism based on the findings. For these reasons, it is crucial to explore ethical considerations around participant protection and ensuring clarity and transparency around how data will be used, where and how it will be shared, and whether it will or can be anonymized effectively [12]. In organizational research, complete anonymity will often not be possible, as individuals may often be easily identified even when network maps are anonymized. Only through addressing these key issues can participants offer truly informed content to participate in network studies. Furthermore, there are implications of non-participation in studies, whereby gaps in network maps may result in an inaccurate representation of how a network is working and this, in turn, means the results may be of lesser value in terms of informing policy and practice in relation to that network.

This is closely linked to one of the most significant challenges in network studies: the problem of missing data. Network studies are more sensitive to missing data than other research methods as they are particularly susceptible to non-response bias, where individuals refuse to answer certain questions or to participate in network studies at all. Respondent burden or asking sensitive questions may increase this risk. Traditionally, it has been contended that a network response rate of 75% is required in SNA for data to be considered reliable [13]. This can be challenging to achieve, particularly in large network or longitudinal studies. 

Given these findings, it is crucial that researchers adopt methods and strategies to encourage and boost response rates to network studies and to generate trustworthy and reliable results that are representative of the true nature of the network. An influential method of reducing the rate of non-response of network members is the building of rapport with respondents before administering network surveys [14]. However, this may be resource-intensive and not always possible in some contexts. Face-to-face contact with the research team can be effective in addressing concerns and clarifying the main ethical issues. Data collection methods may also limit opportunities to build rapport, for instance when surveys are administered online or sent by post and self-administered. Collecting data in group settings or through one-on-one interviews may be a useful alternative in certain situations. As highlighted by Church, response rates can be influenced by social, cultural, and organizational contexts and, thus, researchers need to adopt an approach that is sensitive and appropriate to that context [15].

”Practitioners may be better off choosing an administration method based on factors such as cultural fit and ease of implementation rather than issues of data quality.” [15].

In this case study, we describe the methods undertaken to recruit healthcare managers and clinical leaders to a social network study and share our reflections and learning from the process in the context of the literature on recruitment and sampling in social network studies. 

## 2. Materials and Methods

### 2.1. Purpose of Study

The research we discuss in this case study aimed to recruit senior healthcare managers, executives, and clinical leaders to take part in a network study to explore if collective leadership emerged in a newly established hospital group [16]. In this new system reorganization, 11 hospitals in one region in Ireland became networked and were expected to collaborate and share information to achieve the goals of the wider hospital group in a move towards independence as a hospital trust [17]. 

### 2.2. Context

There were seven hospital groups established in Ireland [17], and one of these groups was the focus of the current study. This hospital group includes eleven hospitals; six are voluntary, and five are statutory hospitals. These two hospital types have different funding and governance structures resulting in very different degrees of autonomy and considerable differences in culture and leadership. Hence, they pose certain challenges for integration and collaborative working. Traditionally, these hospitals had worked mostly independently and without a large degree of collaboration. The hospital group employs more than 10,000 people and services a population of 1.1 million. The current study took place early in the development of the hospital group structure. 

### 2.3. Participants

The hospital group executive management team, as well as Chief Executive Officer or General Manager, Director of Nursing, and Clinical Director in each of the 11 hospitals in the hospital group, were our target participants for exploring the network functioning and, hence, formed the cohort for this study. This sample comprised a total of 44 individuals who were deemed eligible and were invited to participate. Favorable ethical opinion for the research was obtained from the University College Dublin Research Ethics Committee (ref: HREC-LS-16-116397). 

### 2.4. Recruitment Strategies

Given the potential issues regarding involvement in a study exploring collaboration and contact in a newly established hospital group, we carefully considered the most appropriate strategies to inform participants about the study and to reassure them about how the data would be used. To do this, we first presented the aims of the research and introduced social network analysis as a research methodology at presentations to the professional groups included in the study. Each of the 3 distinct professional groups (i.e., CEOs/GMs, Directors of Nursing, and Clinical Directors) typically met on a monthly basis to bring people together across the 11 sites in the hospital group. We delivered a presentation to each of these groups about the research and addressed questions and concerns raised. The first author also arranged one-on-one meetings with those eligible to participate but who could not attend the presentation to inform them about the study and address any queries or concerns. The key during these presentations and meetings was to convey the potential value of the research to the network and how it could help and inform the network’s development and enhance collaboration. 

To encourage participation, we first asked participants to complete an online form to register their consent to participate in the study and to confirm their consent for their names to appear in the network survey, as is consistent with the whole-network roster method. This enabled us to provide further information to participants regarding issues around confidentiality, the potential for identification as part of the network, and confirming that anonymized network maps would be used in all research and reports arising from the research. We also conveyed the risks of opting in, but not taking part in surveys, notifying participants that this could result in incomplete network data and a mischaracterization of their role in the network. We also emphasized that a high response rate from the network would be required for the results to be reliable and informative to the group. 

Despite the high level of interaction and visibility of the research team with participants just before data collection at time point 1, we did not have the capacity or resources to continue this level of engagement through the latter phases of data collection. When new members joined the network, they were emailed the information sheet and consent form along with the presentation that was delivered at the meetings before the launch of the research and a memo of support from the hospital group CEO encouraging their involvement in the study. However, we did not have the opportunity to meet new network members face-to-face about the study again during data collection. We expected that this would be a threat to our response rate, but it was not feasible to continue to engage with the network at that level given our resource constraints and the considerable number of new members joining the network.

Consistent across all time points, the primary researcher emailed invitations to network members at the launch of each of the three network surveys. One reminder was issued by the primary researcher and a further reminder was then issued by the principal investigator (second author), who was familiar and had high visibility, to network members through her involvement in group-level initiatives and on-going research in the hospital group. 

### 2.5. Considerations Regarding Survey Content and Length

One of the principal reasons provided for the low response rate or involvement of healthcare professionals in research is time [4,5]. Given we were asking already very busy senior managers in healthcare to give time to this research, we aimed to minimize the respondent burden through making the online survey as easy and quick to complete as possible. We used the roster method, where all other network members were listed, for two of our network questions on contact and collaborative relationships with others in the network. A third question asked about formal and informal support relationships in the network, but as one would expect fewer of these types of ties in the network, we did not use the roster method and instead asked participants to enter the names of those that they had contacted for support of some kind in the previous month. 

### 2.6. Data Collection

Given this study aimed to chart and map the development of the network over time, we collected data from participants at three time points over the study period (see Table 1). Preliminary results from this study were previously reported in a methods paper based on this research [18]. 

## 3. Results

### Sample and Participation Rates

Our response rates are summarized in Table 1. Of the 44 individuals who met the criteria to take part at the first time point, two opted out, and 35 took part, representing an 80% response rate. However, in the ensuing period, there was considerable disruption and growth in the network with a turnover in roles as well as the expansion of the hospital group executive management team. By the second time point, 52 individuals were eligible to take part and 33 participated, representing a lower response rate of 63%. Finally, by the third time point, there was a 53% response rate from an eligible sample of 55. 

In total, across the three time points, 66 individuals were eligible to take part in the study; this included those eligible at time point 1 and those who were added to or joined the network during the study period.

## 4. Discussion

This paper explored the key considerations in social network study design to maximize the successful recruitment of participants and ensure response rates that enable reliable conclusions about the network that can in turn effectively inform policy. We have elucidated some key issues for researchers to consider in planning network studies for healthcare samples. Among these are the appropriate selection of data sources, consideration in defining network boundaries, sampling and recruitment techniques, and the potential impact of missing or incomplete data. We have used a case study example from previous research exploring collective leadership among senior managers in a newly established hospital network and described our approach to these issues and how they impacted on recruitment and response rates.

One of the key learnings from this study was the considerable benefits of liaising closely with network members before data collection. This high level of engagement had a profound impact on enhancing response rates during the first round of data collection. This is consistent with previous research which advocates the high visibility and interaction of researchers with prospective participants [14]. Through presenting the research aims and overview at hospital group meetings, this served to increase understanding and reinforce the value of the research, as well as offering a platform for researchers to interact with network members to address any concerns or queries they had about the study. This face-to-face contact between the researchers and participants helped to establish the credibility of the researchers in the minds of participants, and this may also have contributed to the high participation rate.

It is also important to highlight that across all time points, a total of 66 individuals were eligible to take part in the study; this included those eligible at time point 1 and those who were added to or joined the network during the study period. Of these 66 individuals, seventeen chose not to participate in any phase of the research. Of the seventeen who did not participate, four left their role (and the network) during the study period, ten were added to the network after the first time point, and three who remained members of the network from the beginning opted to not complete surveys at any point. It is possible that those who joined the network after the first data collection point did not have the same level of awareness of the study. Although all new network members were emailed by the research team and invited to participate on at least two occasions, newer members did not have the same level of contact or familiarity with the research team as those members of the network from time point 1. This echoes the research of Johnson [14] and reflects the value of researchers building rapport with participants of network studies. The high response rate of 80% at the first time point underscores the value of the research team having direct contact with participants in advance of social network studies. The fall in response rates subsequently likely reflects the research team’s inability to maintain that level of engagement and negated the possibility to build rapport with newer network members. This is a limitation of our study, and consequently, we cannot compare recruitment strategies across time points or provide strong evidence of other factors that may have contributed to the fall in response rate. 

One issue experienced in this study, and also possibly an issue for health research in general (though not often discussed), is the high degree of movement of healthcare staff through the system (e.g., frequent restructuring and the establishment of new structures or entities results in managers and leaders being poached or choosing to move to what may be perceived as more challenging or exciting positions). This creates challenges for network researchers to monitor changes and turnover in the first instance and then to effectively recruit new eligible network members to the study. This can place considerable demands on research teams and should be planned for during study design insofar as possible. Longitudinal studies are particularly prone to this challenge, and as a result, researchers may have to develop a plan to deal with missing data and incomplete data sets. Various methods have been proposed to address this issue [19,20,21,22]. However, a detailed review of these options is beyond the scope of this paper. Decisions on the most appropriate course of action will be determined by the study design, research question and details on strength and/or structure of network ties. 

When there is a lower than expected response rate, this has implications for missing data and the reliability of network data when engagement falls below a certain threshold. Although a response rate of 75% is often used as a rule of thumb to indicate sufficient data to draw reliable inferences [13], there has, however, been a valuable body of research exploring the effect of data error and missing data on the validity of network study findings. Researchers have examined the effect of data error on measurement centrality through exploring various kinds of data error, including non-inclusion of nodes and ties [13]. They found that the accuracy of measurement declines predictably as a function of the amount of error introduced. While network centrality measures such as degree and closeness were described as robust where sampling was above 80% of a network, betweenness is more likely to be vulnerable to distortion when sampling is below 70% of the network [23]. Data collection for longitudinal studies can be problematic and may require creativity on the part of researchers to enable and sustain engagement over time. Standard survey-type tools, paper-based or online, are not always practical for large networks, especially when names and network member changes occur regularly during the study period [12].

In networks without clear membership boundaries, or in studies exploring more sensitive research questions, the roster method used in this study would not be appropriate. Snowball or respondent-driven sampling (using participants to identify other network members) has been proposed as one method of recruitment to network studies. This has been criticized for potentially overstating networked connectedness or cohesion, as network members are already connected to those they identify. However, such approaches can be of great value in accessing ‘hidden’ or hard to reach populations, such as drug users or other stigmatized groups. For example, Heckathorn [11] has introduced a method of respondent-driven sampling in which he makes the case for a statistical theory of the sampling process to reliably derive unbiased population indicators [11,24]. The method was applied in a study which sought to recruit drug users in New York [25]. The researchers found that through employing respondent-driven sampling, they successfully recruited 618 drug users from an initial seed sample of just eight. They assert that the method enabled the recruitment of a diverse sample of drug users with a sample profile similar to that of other studies that had recruited drug users in New York using other methods [25].

Despite the many challenges presented by network analysis in healthcare settings, social network analysis has been successfully employed to conduct research in the health sector. However, according to a scoping review, the potential of the method has not yet been fully realized in healthcare [3]. Furthermore, SNA is often used primarily as a descriptive rather than an evaluative tool—another gap that is being addressed through a greater focus on the benefits and unique potential of the methodology and the expanding modelling potential to understand social structure [26].

## 5. Conclusions

For network researchers, human behavior is viewed as embedded in networks of relationships and interactions. Given the increasing multidisciplinary and multiteam working evident in healthcare settings, network methods can offer valuable insights into how relationships may be affecting care, communication, and coordination [7,27]. We strongly advocate and encourage the application of network methods in healthcare settings for the unique insights the approach can offer. Yet, the research design and engagement strategies employed will require careful consideration to maximize the validity and value of the research. Based on our experience, strategies to enhance the visibility of the research team and convey the value and potential impact of the research are crucial to recruitment success and the achievement of high response rates. Where possible, this should include face to face meetings with network members and similar efforts to build rapport that are context-sensitive and feasible [15], with consideration of the resources that may be required to enable this.

## Figures and Tables

**Table 1:** Sample and response rates across time points.

Timepoint	Number Eligible to Participate	Number of Participants (Response Rate)	Non-Responders to Survey
**Time 1 (October 2016)**	42	34 (80%)	4 Clinical Directors, 1 Chief Executive Officer/General Manager (CEO/GM), 1 Director of Nursing and 2 group management team members.
**Time 2 (April 2017)**	54	34 (63%)	5 Clinical Directors, 6 CEOs/GMs, 3 Directors of Nursing and 6 group management team members.
**Time 3 (November 2017)**	55	29 (53%)	7 Clinical Directors, 6 CEOs/GMs, 2 Directors of Nursing and 11 group management team members.

*Note*: Prior to T2, the hospital group management team expanded with the introduction of a transformation program across the group.
